# Supplementary material for: Implementation and Strategies of Community Music Activities for Well-Being: A Scoping Review of the Literature
Source: Int J Environ Res Public Health. 2023 Jan 31;20(3):2606. doi: 10.3390/ijerph20032606 (PMC9914998; doi:10.3390/ijerph20032606)
Supplement: Supplementary file 1 [file ijerph-20-02606-s001.zip › ijerph-2152899-supplementary.pdf]

Supplementary Table S1. Search strings

Pubmed

Search: **((((("community music") OR (singing)) OR (ensemble)) OR (orchestra)) AND (wellbeing)) AND (mental health)** Sort by: **Most Recent**

("community music"[All Fields] OR ("singing"[MeSH Terms] OR "singing"[All Fields]) OR ("ensemble"[All Fields] OR "ensemble s"[All Fields] OR "ensembled"[All Fields] OR "ensembles"[All Fields] OR "ensembling"[All Fields]) OR ("orchestra"[All Fields] OR "orchestras"[All Fields])) AND "wellbeing"[All Fields] AND ("mental health"[MeSH Terms] OR ("mental"[All Fields] AND "health"[All Fields]) OR "mental health"[All Fields])

Translations

singing: "singing"[MeSH Terms] OR "singing"[All Fields]

ensemble: "ensemble"[All Fields] OR "ensemble's"[All Fields] OR "ensembled"[All Fields] OR "ensembles"[All Fields] OR "ensembling"[All Fields]

orchestra: "orchestra"[All Fields] OR "orchestras"[All Fields]

mental health: "mental health"[MeSH Terms] OR ("mental"[All Fields] AND "health"[All Fields]) OR "mental health"[All Fields]

Search: **((choir) AND (wellbeing)) AND (mental health)**

("choir"[All Fields] OR "choirs"[All Fields]) AND "wellbeing"[All Fields] AND ("mental health"[MeSH Terms] OR ("mental"[All Fields] AND "health"[All Fields]) OR "mental health"[All Fields])

Translations

choir: "choir"[All Fields] OR "choirs"[All Fields]

mental health: "mental health"[MeSH Terms] OR ("mental"[All Fields] AND "health"[All Fields]) OR "mental health"[All Fields]

PsychINFO

(((((**Any Field**: "community music") *OR* (**Any Field**: singing)) *OR* (**Any Field**: ensemble)) *OR* (**Any Field**: orchestra)) *AND* (**Any Field**: wellbeing)) *AND* (**Any Field**: mental health)

Search Databases: APA PsycInfo, APA PsycArticles, APA PsycBooks, APA PsycExtra

((**Any Field**: choir) *AND* (**Any Field**: wellbeing)) *AND* (**Any Field**: mental health)

Search Databases: APA PsycInfo, APA PsycArticles, APA PsycBooks, APA PsycExtra

ERIC

((((("community music") OR (singing)) OR (ensemble)) OR (orchestra)) AND (wellbeing)) AND (mental health)

((choir) AND (wellbeing)) AND (mental health)

Web of Science

(ALL=((((("community music") OR (singing)) OR (ensemble)) OR (orchestra)) AND (wellbeing)) AND (mental health)))

ALL=((((choir) AND (wellbeing)) AND (mental health)))

Scopus

TITLE-ABS-KEY (((((("community music") OR (singing)) OR (ensemble)) OR (orchestra)) AND (wellbeing)) AND (mental AND health))

TITLE-ABS-KEY (((choir) AND (wellbeing)) AND (mental AND health))

The Cochrane Library

(((((("community music") OR (singing)) OR (ensemble)) OR (orchestra)) AND (wellbeing)) AND (mental health)) (Word variations have been searched)
